# Virtual Surgical Planning Computer-aided Design-guided Osteocutaneous Fibular Free Flap for Craniofacial Reconstruction: A Novel Surgical Approach

**DOI:** 10.7759/cureus.6256

**Published:** 2019-11-29

**Authors:** Matt Davey, Niall M McInerney, Tom Barry, Alan Hussey, Shirley Potter

**Affiliations:** 1 Plastic, Aesthetic and Reconstructive Surgery, Galway University Hospitals, Galway, IRL; 2 Oral and Maxillofacial Surgery, Galway University Hospitals, Galway, IRL; 3 Plastics, Aesthetic and Reconstructive Surgery, Galway University Hospitals, Galway, IRL

**Keywords:** plastic surgery, oncological surgery, reconstructive surgery, oral cancer, oral surgery, computer-aided design, maxillofacial prosthetics

## Abstract

Advancements and increased availability of radiological services have revolutionised our approach to oncological and reconstructive surgical practice. With an increasing demand for accuracy in diagnosis and improved oncological outcome, the requirement for precise application of radiological tools and the exploration of novel software has developed. This has led to the evaluation of modern technologies such as computer-aided design to enhance reconstructive surgery.

Mandibular reconstruction following oncological resection using an osteocutaneous fibular free flap is now considered to be the gold standard reconstructive surgical approach, as this approach provides more satisfactory outcomes for both patients and reconstructive surgeons. Recent years have seen Irish reconstructive surgeons introduce computer-aided design pre-operative planning to operating theatres as means of improving cosmetic, functional and oncological outcome, yet the detailed, complex planning required pre-operatively is not well described.

Herein, the purpose of this article was to demonstrate the precision and accuracy of virtual surgical planning computer-aided design (VSP-CAD) as a modern surgical approach to craniofacial reconstruction following surgical resection of an American Joint Committee on Cancer stage 4 oral carcinoma.

## Introduction and background

**Figure 1:**
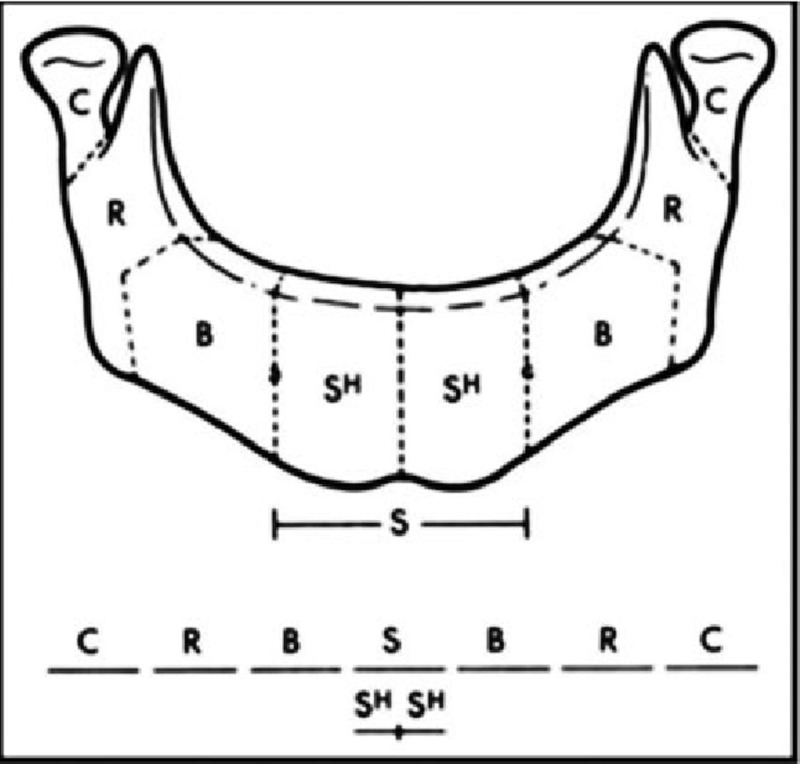
Urken classification of mandibulectomy defects

Oral cancers account for 4% of all cancers in the western world [1]. Squamous cell carcinoma is the most prevalent type of carcinoma affecting the buccal cavity, and mandibular involvement is seen in 49% of cases [2]. Neoplastic facial changes are often disfiguring, and furthermore en-bloc oncological resection can lead to devastating cosmetic and functional deficits with resultant psychological, physical, functional and nutritional effects. Craniofacial reconstruction in such cases poses unique challenges due to the three-dimensional configuration of the proposed construct and the critical importance of restoring speech, swallowing, mastication and symmetrical facial contour. Additionally, reconstruction results are often inconsistent and learning curve dependent, making pre-operative planning difficult for reconstructive surgeons [3]. Classically, mandibular reconstruction using free osteocutaneous fibular flaps was first described by Hidalgo in 1989 [4], and classified by Urken (as per Figure 1), and has until recently relied mainly on the use of surgical trial-and-error and 2D imaging modalities.

## Review

**Figure 2:**
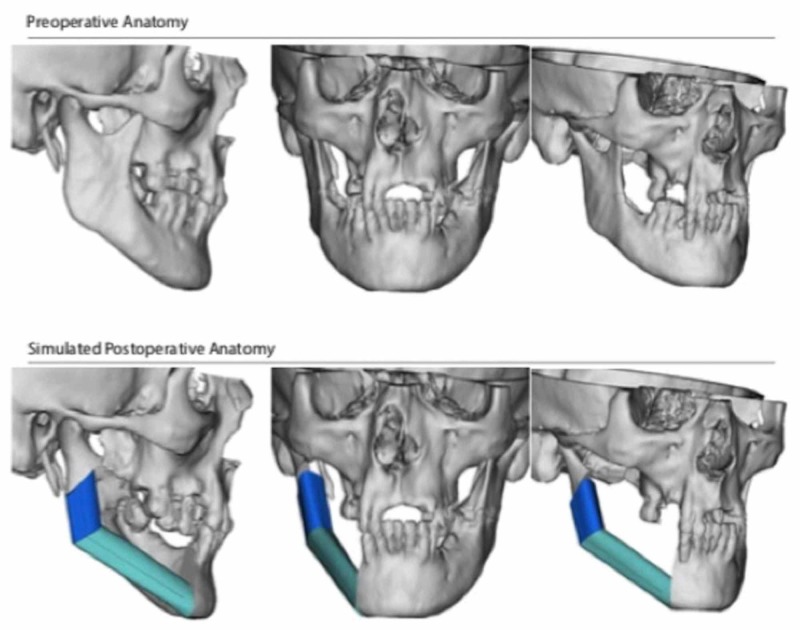
(A) Preoperative anatomy, and (B) simulated postoperative anatomy based on patient specific CT imaging (courtesy of VSP®)

**Figure 3:**
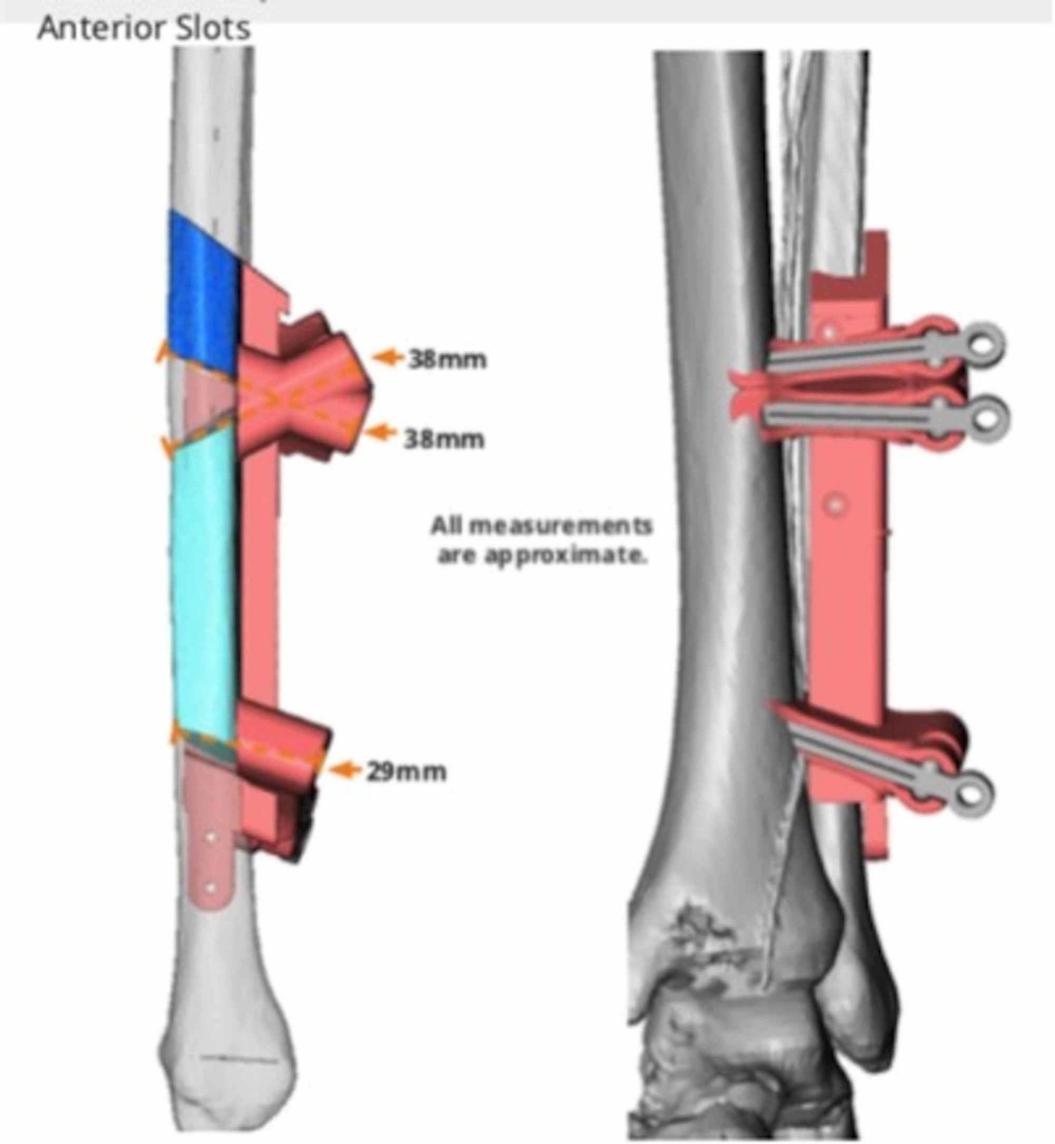
Anterior view of planned fibular osteotomy (courtesy of VSP®)

**Figure 4:**
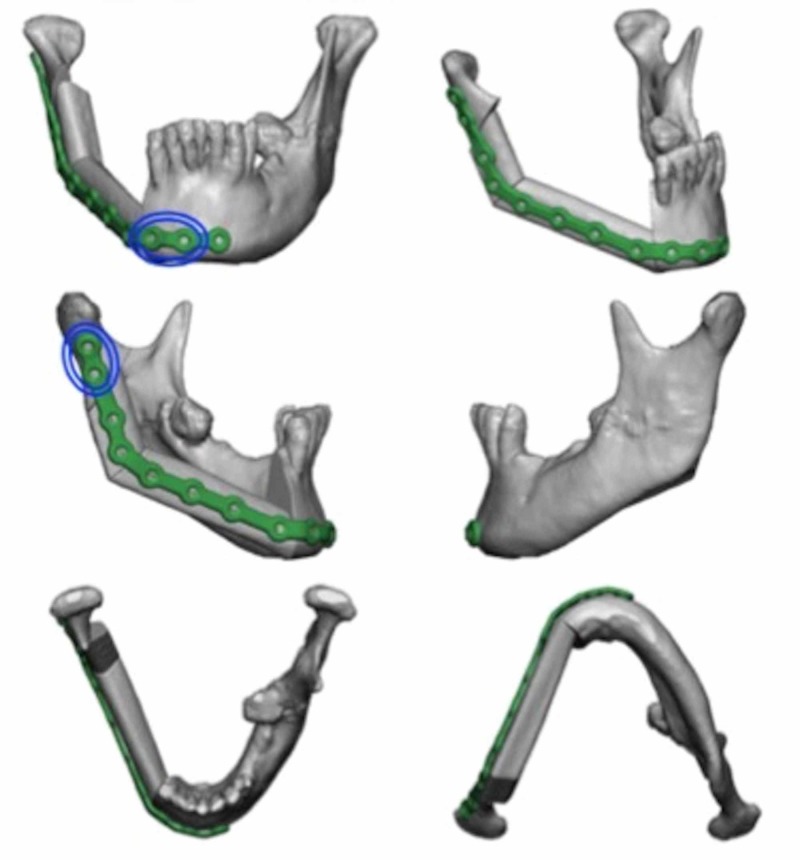
Mandibular reconstructive guide plate with predictive marking holes marked in blue (courtesy of VSP®)

**Figure 5:**
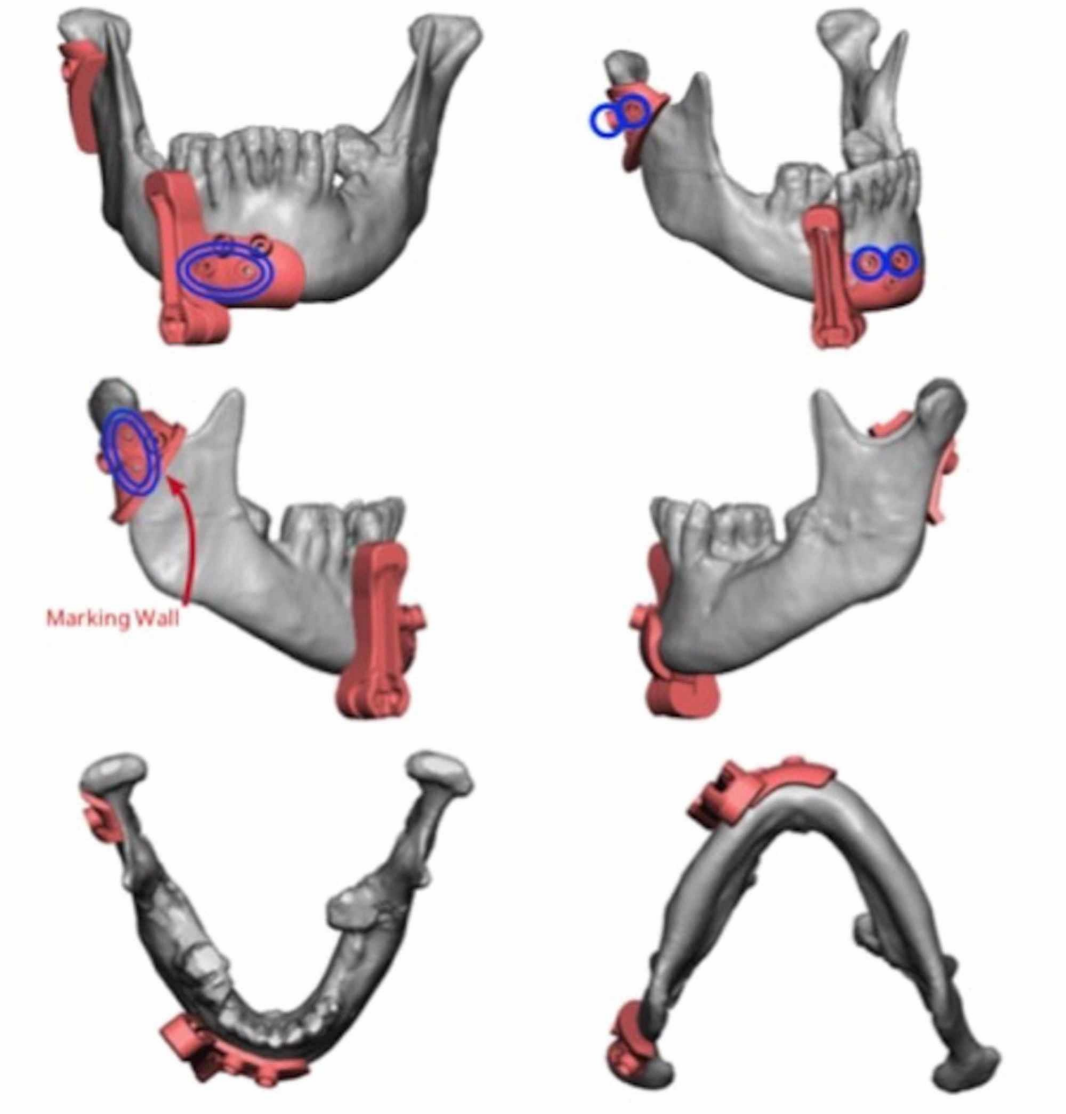
Planned placement of mandibular cutting plates for adequate resection with clear margins (courtesy of VSP®)

**Figure 6:**
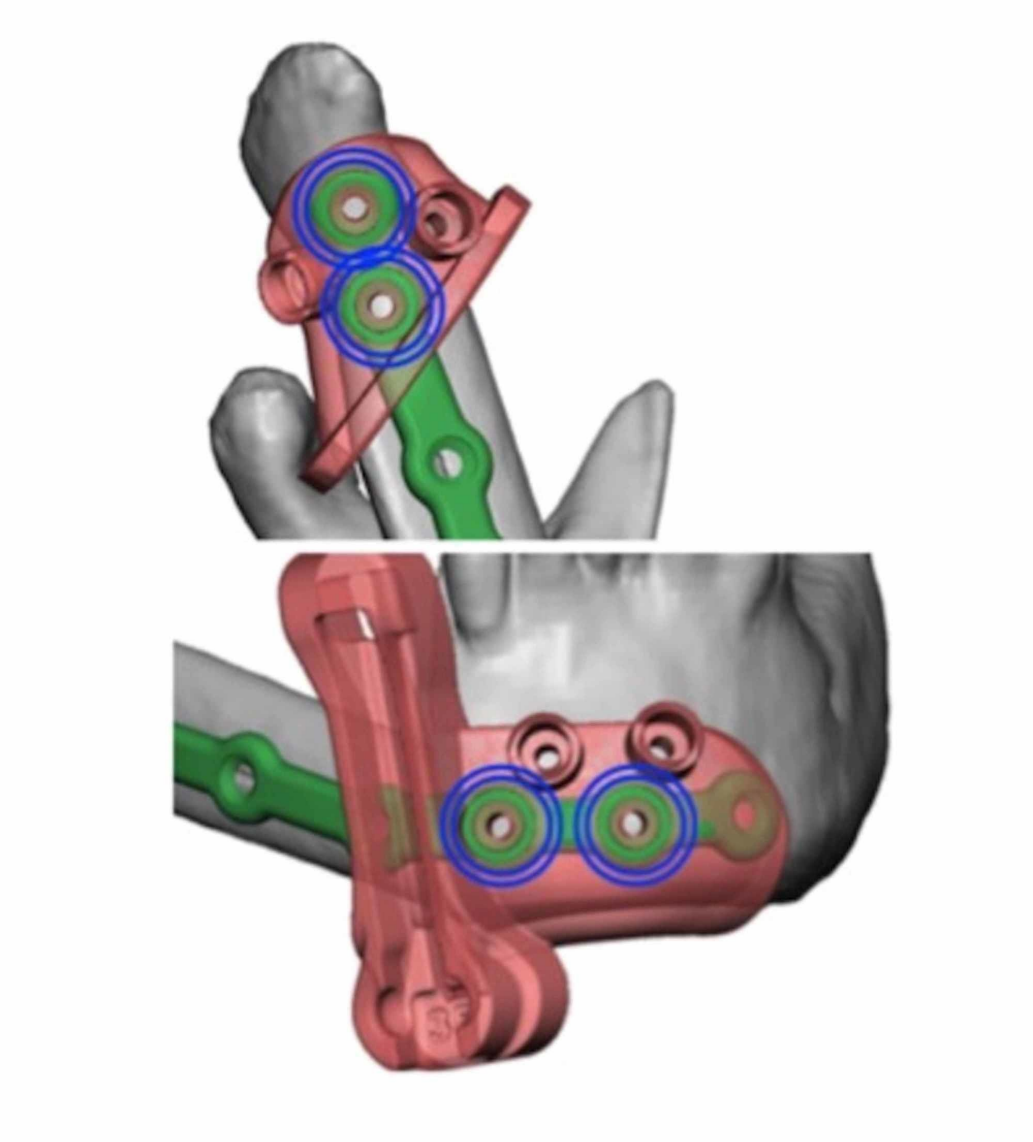
Overlapping cutting and reconstructive guide plates, with predictive marking holes in blue (courtesy of VSP®)

Virtual surgical planning and computer-aided design (VSP-CAD) are novel technologies that present advantages in the planning and execution of such challenging traumatic and oncological craniofacial reconstructions [5]. As with many modern surgical techniques, VSP-CAD follows a remarkably complex and sophisticated pre-operative course: VSP-CAD begins with the acquisition of a 3D rendered 64 slice high-resolution CT scan of the patient’s craniofacial skeleton (Figure 2) and an angiographic CT scan of the lower leg as donor site study for bone and vessels. A web meeting between oncological surgeons, reconstructive surgeons and biomedical engineers sequentially determines a number of pre-planned operative factors: the amount of bone resection to allow an adequate oncological margin, the laterality of fibular free flap to be used (Figure 3), the number and obliquity of osteotomies required to shape the fibula to fill the exact mandibular defect post-osteotomy. Virtual predictive plans of customised reconstructive plate in-setting (Figure 4), screw-hole position (Figure 5), and cutting-plate position in relation to the remaining mandible and craniofacial skeleton (Figure 6) are all derived from the pre-operative multidisciplinary web meeting.

A number of reported functional benefits of VSP-CAD include increased reconstructive accuracy, more efficient use of theatre time, and an overall reduction in flap ischemia time [6,7]. Subjective benefits of VSP-CAD include improved patient and surgeon satisfaction being reported [8]. Furthermore, precise preoperative planning and communication between the oncologic and reconstruction teams have previously been limited by the lack of accurate data regarding the anatomy of the lesion, precise margins of resection, and anatomy of the graft recipient site [9,10]. Prior to VSP-CAD application, such obstacles to efficiency only revealed themselves in the operating room mid-procedure, when surgeons had to depend on two-dimensional imaging modalities such as X-ray to make three-dimensional estimates. Thus, for a procedure requiring a high degree of precision for optimal orthognathic and aesthetic outcomes, craniofacial reconstructive success has historically been hindered by prolonged intraoperative time and suboptimal reconstructions [7,9]. VSP-CAD seems to offer a resolution to increased multidisciplinary communication, enhanced cooperation between surgical teams, better pre-operative planning, and the ability to customise models to patient’s individual characteristics, whilst offering potential for considerable intra-operative time saving [3]. With these factors being considered, VSP-CAD seemingly offers significant benefits for use in complex oncologic osseous head and neck reconstruction by fostering the above surgical factors.

Additionally, as VSP-CAD offers the resecting surgeon with pre-operative three-dimensional CT visualisation of the lesion margins and a comprehensive plan from the reconstructive team, the surgeon may be more inclined to plan liberal resection margins initially, thus potentiating decreased local recurrence rates and intraoperative time [3]. Similarly, reconstructive planning may be better performed by reconstructive surgeons with advance knowledge of the resection plan. Technological refinements in the VSP-CAD interface have become progressively more user-friendly for application by both the resecting and reconstructive surgeons, allowing adoption of this technology and coordinated pre-operative planning to increase in more centres worldwide [5]. For the above reasons, VSP-CAD is rapidly gaining traction for a range of surgical applications ranging from trauma to oncologic reconstruction, and seems to have a logical role in future surgical planning.

## Conclusions

We propose that using virtual surgical planning and computer-assisted design for craniofacial reconstruction after ablative oncological surgery significantly improves the functional and cosmetic results, allowing for accurate recovery of function and facial contour. This novel surgical technique also has the potential to decrease length of time taken in Irish operating rooms to perform craniofacial surgical reconstruction, without compromising results.

## References

[1] Markopoulos AK (2012). Current aspects on oral squamous cell carcinoma. Open Dent J.

[2] Rogers SN, Devine J, Lowe D, Shokar P, Brown JS, Vaugman ED (2004). Longitudinal health-related quality of life after mandibular resection for oral cancer: a comparison between rim and segment. Head Neck.

[3] Antony AK, Chen WF, Kolokythas A, Weimer KA, Cohen M (2011). Use of virtual surgery and stereolithography-guided osteotomy for mandibular reconstruction with the free fibula. Plast Reconstr Surg.

[4] Hidalgo DA (1989). Fibula free flap: a new method of mandible reconstruction. Plast Reconstr Surg.

[5] Sharaf B, Levine JP, Hirsch DL, Bastidas JA, Schiff BA, Garfein ES (2010). Importance of computer-aided design and manufacturing technology in the multidisciplinary approach to head and neck reconstruction. J Craniofac Surg.

[6] Chim H, Wetjen N, Mardini S (2014). Virtual surgical planning in craniofacial surgery. Semin Plast Surg.

[7] Resnick C, Inverso G, Wrzosek M, Padwa BL, Kaban LB, Peacock ZS (2016). Is there a difference in cost between standard and virtual surgical planning for orthognathic surgery?. J Oral Maxillofac Surg.

[8] Modabber A, Legros C, Rana M, Gerressen M, Riediger D, Ghassemi A (2012). Evaluation of computer-assisted jaw reconstruction with free vascularized fibular flap compared to conventional surgery: a clinical pilot study. Int J Med Robot.

[9] Efanov J, Roy A, Huang K, Borsuk D (2018). Virtual surgical planning: the pearls and pitfalls. Plast Reconstr Surg Glob Open.

[10] Herford A, Miller M, Lauritano F, Cervino G, Signorino F, Maiorana C (2017). The use of virtual surgical planning and navigation in the treatment of orbital trauma. Chin J Traumatol.

